# A Survey of Recent Patents in Engineering Technology for the Screening, Separation and Processing of Eggshell

**DOI:** 10.3389/fbioe.2021.677559

**Published:** 2021-05-04

**Authors:** Tamer A. E. Ahmed, Manar Younes, Ling Wu, Maxwell T. Hincke

**Affiliations:** ^1^Department of Cellular and Molecular Medicine, Faculty of Medicine, University of Ottawa, Ottawa, ON, Canada; ^2^School of Nutrition Sciences, Faculty of Health Sciences, University of Ottawa, Ottawa, ON, Canada; ^3^Department of Innovation in Medical Education, Faculty of Medicine, University of Ottawa, Ottawa, ON, Canada

**Keywords:** eggshell, patents, screening, collection, separation, processing

## Abstract

The chicken egg is a well-known complete food of human daily consumption which serves as a cost-effective, high-quality nutrient resource. About 30% of table eggs are directed to breaker plants in developed countries, leading to the generation of substantial eggshell (ES) waste, which is increasingly explored for potential value-added applications. The number of patents describing ES-based applications has increased dramatically in recent years. This review provides insight into the most recent patents published between 2015 and 2020, with focus on different engineering technologies for the screening, separation, and processing of ES. Screening technologies include detection of ES surface spots and glossiness, ES cracks, and mechanical properties, along with identification of chicken breed and enumeration of surface bacterial count. Collection and separation technologies describe separation strategies of ES from egg white (EW), egg yolk (EY), liquid egg, eggshell membrane (ESM), hatchlings, and cooked egg. Separation of ES from liquid eggs utilizes gravity, rotational forces, or air pressure. Processing of ES involves washing and sterilization along with cutting, crushing, and pulverization technologies that enable the collection of ES suitable for value-added applications. In addition, ES carving (mechanical and laser) opens up the realm of artwork and decoration. Furthermore, intact ES can be utilized for food serving. The exponential increase in innovative screening, separation, collection, and processing technologies reflects industrial interest to upscale low-value ES waste material, and is a first crucial step in the emergence of advanced technologies that exploit the biomedical, chemical, engineering, and environmental applications for ES.

## Introduction

**FIGURE 1:**
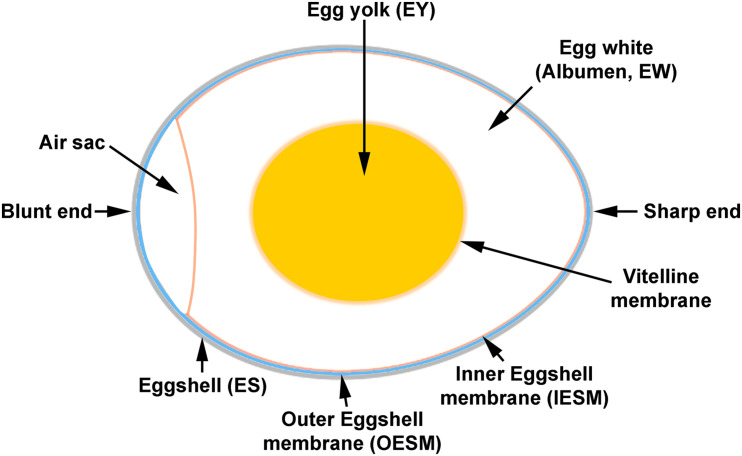
Schematic presentation of a chicken egg. Eggshell (ES), outer eggshell membrane (OESM), inner eggshell membrane (IESM), egg white (albumen, EW), vitelline membrane, and egg yolk (EY). The air sac separates the OESM from IESM at the blunt end.

The calcareous avian egg represents the most advanced amniotic egg in oviparous vertebrates and comprises multiple layers that originate from different oviduct segments (Hincke et al., 2012). The average chicken egg (60.9 g) consists of 10% eggshell (including shell membrane), 63% albumen (egg white), and 27% yolk (Hincke et al., 2012; Nimalaratne and Wu, 2015; Laca et al., 2017; Figure 1). The chicken egg is known as the most complete human food, and is a vital feature of everyday human consumption that serves as a low-cost and high-quality nutrient resource (Ahmed et al., 2019a).

**FIGURE 2:**
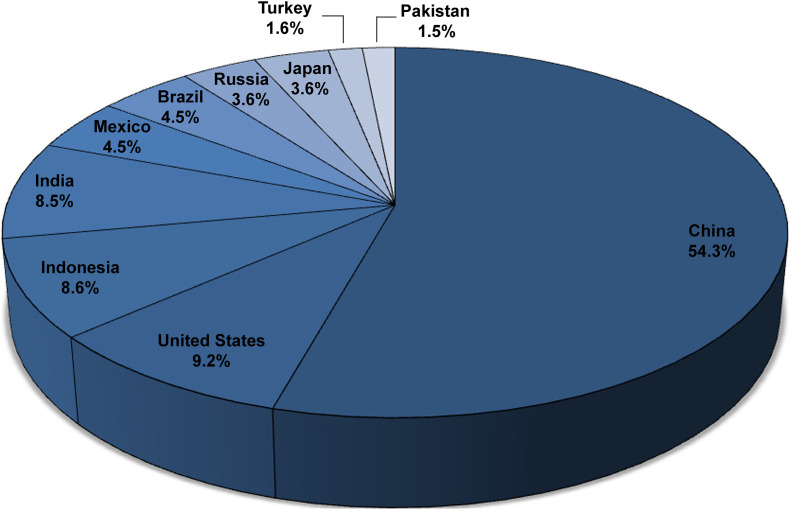
Annual egg production by top 10 countries in 2019 (FAO, 2020).

**FIGURE 3:**
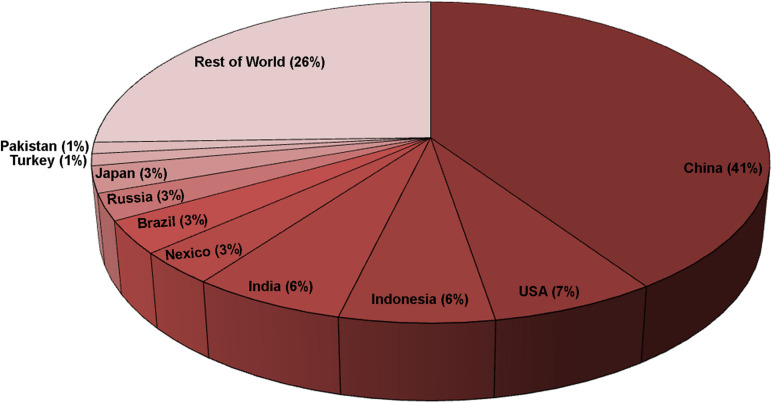
Eggshell waste generated by breaking plants in the top 10 countries in 2019 (FAO, 2020). Calculated as a percentage of the world total (29.7 Megatonnes). Approximately 30% of the produced eggs are diverted to the breaking plants (Ahmed et al., 2019b; Owuamanam and Cree, 2020; Kulshreshtha et al., 2020). ES and ESM together constitute about 10% of the egg weight (Hincke et al., 2012; Nimalaratne and Wu, 2015; Laca et al., 2017).

The ten leading egg-producing countries are (in order): China, the United States, Indonesia, India, Mexico, Brazil, Russia, Japan, Turkey, and Pakistan, which together contributed approximately 75% of total global egg production (∼1,652 billion eggs, 99 million tonnes) in 2019 (FAO, 2020; Figure 2). About 30% of table eggs are diverted to breaker plants in developed countries to produce various egg products and generate massive quantities of eggshell waste (Tsai et al., 2006; Ahmed et al., 2019b; Kulshreshtha et al., 2020; Figure 3). Numerous patents have been disclosed to utilize ES waste for potential value-added applications; however, ES is still undervalued (Cordeiro and Hincke, 2011; Ahmed et al., 2019a).

**FIGURE 4:**
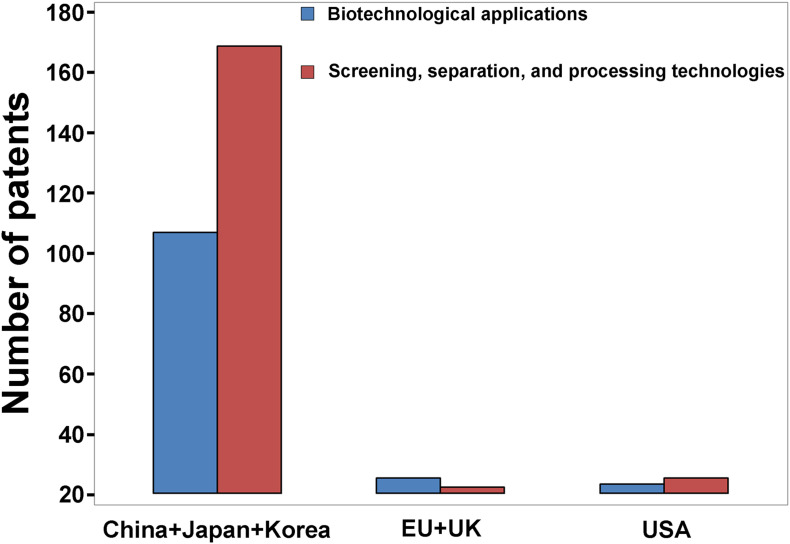
Geographic distribution of ES patents between 2015 and 2020 amongst different countries, showing the dramatic output from Asian countries (China, Japan, South Korea, and Taiwan) (Data derived from the Google patent research engine).

For this review, the Google patent research engine was utilized to identify global trends in patents pertaining to ES over the past century, using “eggshell” as a keyword. The overall patent numbers, annual numbers, and geographical distribution of ES-based patents were discussed in our companion article (Ahmed et al., unpublished). We observed that there was a large increase in the last two decades due to a remarkable increase in the number of patents originating from Asian countries including China, South Korea, Japan, and Taiwan. Between 2015 and 2020, 257 patents related to ES applications of avian egg were disclosed. About 60% of these patents (158 patents) describe engineering technologies for the screening, separation, and processing of ES (the focus of this article), while around 40% describe biotechnological applications of ES (discussed in our companion article, Ahmed et al., unpublished). Approximately 88% (biotechnological applications) and 94% (engineering technologies) of these patents issued from Asian countries, including China, South Korea, Japan, Philippines, and Taiwan, while less than 5% of patents were disclosed in Europe and the United States (Figure 4). This review specifically focuses on the recent patents published between 2015 and 2020 for the different engineering technologies for ES screening, washing, sterilization, separation, and processing (Table 1).

**TABLE 1:** Roadmap for the review.

1. Introductions		
2. Eggshell Screening Technologies	2.1 Screening of eggshell surface spots and glossiness 2.2 Identification of chicken breed and enumeration of surface bacteria 2.2 Screening of eggshell cracks in intact eggs 2.2 Screening based on eggshell mechanical properties	
3. Eggshell Screening Technologies	3.1 Separation of eggshell from egg white and egg yellow 3.3 Separation of eggshell from liquid egg 3.3 Separation of eggshell from eggshell membrane 3.3 Separation of eggshell from hatchlings or cooked eggs	3.2.1 Separation of liquid egg using rotational forces 3.2.2 Separation of liquid egg using gravity 3.2.3 Separation of liquid egg using air pressure 3.4.1 Separation of eggshell in the broiler sector 3.4.2 Separation of eggshell from cooked eggs
4. Processing Of Eggshell	4.1 Eggshell washing and sterilization 4.4 Artistic carving of eggshell 4.4 Eggshell for artwork and decoration 4.4 Eggshell cutting 4.4 Eggshell crushing 4.4 Eggshell powder 4.4 Intact eggshell	Mechanical carving Laser carving Other carving strategies
5. Conclusion and Prospects		

## Eggshell Screening Technolgies

The chicken egg is a popular and nutritive food in the human diet. However, ES is susceptible to breakage during routine handling and transportation, resulting in economic losses to the egg industry. Most importantly, clean and intact eggs are essential to maintain egg quality for consumer satisfaction and for food safety. Therefore, this industrial sector has invested heavily in the development of crack detection technologies and improved handling systems to minimize ES breakage (Bain et al., 2006). In the last 5 years, various patented inventions have been developed to monitor ES quality, including detection of cracks and black spots, determination of breaking strength, assessment of bacterial contamination, and ES surface glossiness. Quality control of these characteristics is essential for food safety and maintenance of consumer satisfaction.

### Screening of Eggshell Surface Spots and Glossiness

ES surface stains can be inspected using an online system involving transfer roller, industrial camera, Image acquisition case, illumination source, photoelectric sensor, programmable logic controller, and computer. The industrial camera captures the image of the illuminated egg as it transits the image acquisition case; surface stain locations are detected by image analysis (Duan, 2018). ES surface colored spots can be detected using a light scanner. A triangular prism-shaped refitting device scans the ES surface using a Uniscan LA2000 purple light scanner, which has the advantage of quick scanning speed with good quality. The refitting device, in combination with the scanner, enables the detection of ES surface dark spot numbers and their area via image analysis (Ye et al., 2020). Another patent describes a modification of a vancometer (Glossmeter) to measure ES surface glossiness, which is a good indicator of freshness and quality. A typical vancometer measures the glossiness of either a flat object or an object with a small radius, in which the vancometer measuring slit size is very small, and the slit-holding surface is rigid. Therefore, the assessment of glossiness of a spherical object, such as an egg, is technically challenging. This invention has a larger measuring slit size, with an elastic slit-holding surface to accommodate the curved egg surface (Ye et al., 2018).

### Identification of Chicken Breed and Enumeration of Surface Bacteria

A technique has been disclosed to identify the chicken breed of origin of an egg, which involves extracting maternal DNA from the ES. A small sample of ES is cleaned, powdered, and mixed with lysis buffer to extract and purify genomic DNA. The purified DNA is amplified using polymerase chain reaction (PCR) with breed-specific primers. DNA can be extracted from as little as 100 mg of ES powder with high reproducibility and without compromising egg integrity (Fan et al., 2019). In another disclosed patent, the ES surface total bacterial count was measured efficiently using microcalorimetry. The calorimetric profile of viable bacterial strains is measured with a TAM III multichannel micro-calorimeter. The determined total calorific value of the tested sample, based on a standard growth curve, is used to obtain the total number of bacterial colonies (Guo et al., 2016).

### Screening of Eggshell Cracks in Intact Eggs

Strategies for the detection of ES cracks include improved visualization of the egg surface. One recently patented intervention assists poultry farm workers in detecting ES cracks to accelerate the screening process. The inspection device is composed of an opaque rectangular body with a square groove that fits a transparent carrier module. Eggs placed in the carrier module are illuminated to quickly identify cracked eggs (Zheng P., 2019). An alternative strategy involves the staining of the ES surface with food-grade pigment to facilitate the visual inspection and exclusion of cracked eggs. The intact stained eggs are then cleaned to remove the pigment from the ES surface (Zhang and Chen, 2018; Zhang and Chen, 2019). Cracks can also be detected using image processing software following analysis of a series of acquired grayscale images that cover the entire egg surface. Comprehensive pixel scanning and analysis of the grayscale image series using dynamic programming enables the creation of continuous curves that indicate the presence or absence of surface cracks (Duan and Wang, 2018).

Various patents describe the utilization of sound waves (i.e., resonance-dependent) to identify the location of surface cracks using intact eggs as a standard. Signals generated after egg tapping are recorded by a microphone and analyzed using Fast Fourier transform (FFT) spectrum analysis. The resonance frequency and amplitude of cracked eggs are different from those of an intact egg (Zheng J., 2019). Similarly, the audio signal produced by a small ball briefly bouncing on the ES surface is analyzed to determine whether ES surface is intact (De Ketelaere et al., 2018). Another approach detects ES microcracks using a mechanical rotating module with a tapping hammer, microphone, and audio spectrum analyzer. The tapping hammer vibrates the ES surface, and the microphone collects sound wave signals. Spectrum analysis allows detection of the slight differences between intact eggs and those with ES defects (Zheng, 2018). Multi-station acoustic response signal analysis can also detect ES cracks. The egg surface is exposed to percussion rod excitation at different excitation positions, and the generated sound waves undergo multi-signal analysis to detect the presence of cracks (Chen C. et al., 2019). In another approach, three vibration acceleration sensors and one acoustic pulse sensor are used to collect ES surface information at four different locations. The collected data is processed to establish a discrimination model to determine whether an egg is intact or cracked (Lin et al., 2015).

Alternatively, the egg surface is subjected to deformation by vibration resonance, and crack information is obtained using a laser beam to scan the vibrating ES surface. The scanning and reflection signals are processed using a self-mixing laser vibrometer (SMLV) to obtain a mixed signal with amplitude (van Wegen et al., 2017). ES crack inspection can be based on a hollow knocking body that encloses a stress emitter and ES state inspection device. The stress emitter produces light signals according to the deformation that takes place after hitting the egg surface at the blunt and sharp ends. The intensity of the generated light is analyzed by the ES state inspection device to determine whether the ES is intact (Fujitani, 2020).

### Screening Based on Eggshell Mechanical Properties

ES strength is another indicator of egg quality. An ES strength measuring invention comprises a base, a pressure applying device, a first detecting device (resistive touch screen), and a second detecting device (pressure sensor). The egg is placed between the first detection device located at the base and the second detection device attached to the pressure-applying device. The first detection device is utilized to detect the egg contact surface area, while the second detection device detects the pressure (F) at which the eggshell breaks (Zhang et al., 2019b). Alternatively, a portable ES strength analyzer is composed of a volumetric cup fixed on the top of pressure lid, egg socket, and a base. The egg is held between the pressure lid and the egg socket while water is gradually added to the volumetric cup until egg breakage occurs. The volume of added water is proportional to ES strength (Long and Yuan, 2019). In contrast, the ES elasticity parameters of ES at the equatorial region can be measured accurately and non-destructively. The egg is vertically enclosed between two plates with the sharp end facing up. Displacement force is then applied to the sharp end, and the strain recorded vertically and horizontally at the egg equatorial region via foil-gauges in order to determine the elasticity modulus (E) and Poisson’s ratio (μ) (Zhang et al., 2018).

## Collection and Separation Technolgies for Eggshell Waste

In developed countries, about 30% of shell eggs are diverted to breaker processing plants to produce liquid eggs (Cordeiro and Hincke, 2011; Ahmed et al., 2019a). The ES and associated ESM are approximately 10% of egg weight and are a substantial byproduct (Laca et al., 2017). ES collection involves the separation of liquid egg contents (yolk, white) and the diversion of the ES and its firmly attached ESM for further processing or to waste collection (Ahmed et al., unpublished). Numerous inventions have been patented for ES separation, including automatic and manual approaches. In the following section, recent patents pertaining to the separation of ES are discussed.

### Separation of Eggshell From Egg White and Egg Yellow

A number of processes for simultaneous separation of egg white (EW) and Egg yolk (EY) from ES have been recently patented (Liu, 2016; Lu and Zhao,2016a,b; Zhou,2017a,b; Gu et al., 2018; Liu, 2020). An automatic ES breaking device has been disclosed for the field of food processing, which constitutes a rotating seat, egg-bearing plate and a breaking mechanism based on a breaking knife. The eggs on the egg-bearing plates are pressed against the shell knife until a crack is formed at the bottom of the ES. EY and EW are then separated and collected using a vibration-based mechanism (Liu, 2020). Another automatic egg-breaking device separates ES, EW, and EY efficiently. Briefly, the separation line includes egg-feeding pallet, egg catching device that advances by a transmission chain (driven by a power shaft) to a breaking device. Eggs are broken using a fast-moving blade and the separation of EW from EY is mediated by a diversion groove. EW flows to the EW outlet via the diversion groove, while EY is retained and flows to the EY outlet. Both outlets are located at the lower part of the blade holder. ES accumulates at the ES outlet located at the rear of the device (Lu and Zhao,2016a,b). Another patented process separates ES, EY and EY using a tilted rotating drum that contains numerous leakage holes. Broken eggs slide into the rotating drum and EW sinks quickly to the bottom of the vibrating separation bucket (connected to the lower end of the rotating drum). Slow rotation of the drum causes ES to adhere to the inner drum wall, and it accumulates into the ES box at the rear end of the drum (Zhou,2017a,b). A shaft-dependent device for the synchronous breaking of ES and separation of EW from EY consists of an egg-beating module located over EW and EY separation modules that facilitate the collection of egg contents in an egg container. A rotary mechanism permits EW to flow into the EW collection tank through the EW separation hole located at the egg container bottom, while EY is retained. Tilting the egg container pours accumulated EY into the EY collection tank (Gu et al., 2018). A simple invention describes a raw stainless steel ES breaking device consisting of two connected semi-ellipsoidal shells, with an upwardly ES breaking blade arranged at the joint, and EW hole. After ES is broken by the blade, EW and EY flow to the semi-ellipsoidal shells, where the EY flows out through the EW hole, while the EY is retained. This invention claims to have advantages of simple structure, low production cost, and fast ES breaking speed, with little contamination of egg liquid by ES fragments (Liu, 2016).

### Separation of Eggshell From Liquid Egg

Various strategies have been patented for the automated or manual separation of ES from liquid egg, which utilize rotational forces (Wang and Cheng, 2018; Liu Z. et al., 2019; Wen,2019a,b), gravity (Bian,2015a,b, 2016a,b; Luo,2016b,c; Xia, 2017; Liang et al., 2018; Sakino, 2018; Gong, 2019; Hua, 2019; Li, 2019; Yin and Yin, 2019) or air pressure (Wang,2016a,b; Wang et al., 2019; Yang, 2016; Wu, 2017a; Yao et al., 2017; Dong et al., 2018; Wu, 2018; Yan et al., 2018; Zhang M., 2018).

#### Separation of Liquid Egg Using Rotational Forces

**FIGURE 5:**
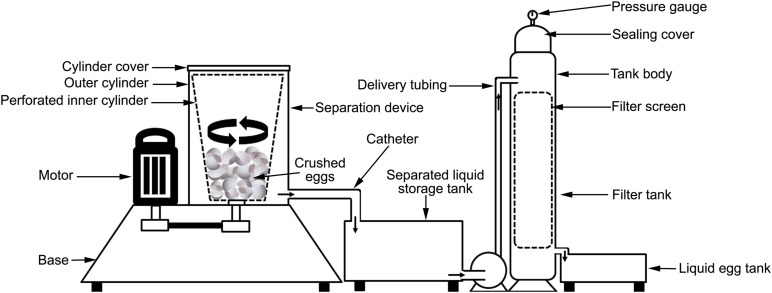
Schematic presentation of patent CN209564036U describing a method to separate ES from the liquid egg.

A patented process describes a drum-type ES/liquid separator consisting of a rapidly rotating perforated “strainer” bucket. Intact eggs are dropped via a funnel-shaped feed inlet into the strainer and break against the rapidly- rotating bucket wall. Centrifugal forces drive the egg liquid through the strainer drain holes into an outlet tube, while the crushed ES is retained in the bucket (Wen, 2019a). An updated drum-type ES/liquid device is patented where eggs are placed first in multiple filter bags which are then beaten against the baffle plates of rapidly-rotating bucket wall. Liquid egg then flows to the outlet bucket, while ES is retained in the filter bags (Wen, 2019b). A similar invention discloses an egg liquid separation device consisting of a separator unit connected to a liquid egg storage tank, filtering tank, and liquid egg slot. The separator unit is composed of outer cylinder, inner perforated cylinder, and a cover. Following egg crushing, the egg liquid is strained by high-speed rotation of the motor-driven perforated inner cylinder, and then flows into the storage tank. Separated liquid egg material is then pumped into the filtering tank to filter out ES fragments and collected in the egg liquid slot (Liu Z. et al., 2019; Figure 5). A related liquid egg separation strategy is patented where the eggs are introduced via a feeding hopper to a centrifugal barrel, where they are broken by the action of a rotating rod and spiral blades. Centrifugal forces drive egg liquid through the strainer to the egg liquid outlet, while the ES accumulates at the ES outlet via the staged rotation of the spiral blades (Wang and Cheng, 2018).

#### Separation of Liquid Egg Using Gravity

A patent reports an effective approach to separate liquid egg from ES, where egg is dropped via a feed inlet into an angled crushing auger containing a motor-driven crushing spiral shaft. Liquid egg flows into the liquid egg collecting box through a strainer at the auger lower end under the effect of gravity, while rotation of the spiral shaft transports the ES fragments upwards to the drying unit via a discharge nozzle. The dried ES undergoes a pulverization step before collection (Li, 2019). A similar patent describes a liquid egg separation device with an optimized recovery rate, which is based on a helical blade that crushes eggs and directs the flow of liquid egg to a discharging tube. In addition, the slow spiral motion facilitates the adherence of ES to the blade surface (Xia, 2017). Another gravity-dependent separation invention sequentially arranges eggs on an elevated feed hopper with oval leak holes in a carrier plate connected to a conveyer belt. During the carrier plate movement, eggs are broken using a shaft-connected clapper with the subsequent dropping of egg liquid into a discharge bucket through the leak holes. ES is carried forward on the carrier plate to a collection receptacle. The feeding of eggs to the carrier plate, collection of egg liquid and accumulation of ES all depend on gravity (Hua, 2019). Egg contents can also be collected after drilling a hole through an otherwise intact ES. Such a device is composed of a big pipe, splenium, pointed insert link connected to a compression spring, U-shape egg container with a bottom hole, and collection container. Eggs are placed in the U shape egg container, fixed inside the big pipe, then punctured from top to bottom by the pointed link via pressing the splenium. The egg contents drain to the collection container via the U-shape egg container bottom hole (Luo,2016b,c). To promote hygienic and smooth egg breaking along with separation of egg contents, a simple folded stainless steel device was patented. Striking an egg against the apex of the fold allows the egg contents to flow down the sloping side for collection (Sakino, 2018).

Some manual and facile ES separators have been patented to ensure the collection of hygienic egg contents with a high recovery rate. A kitchen ES stripper was patented to separate ES manually using a cutting egg-shaped clamping device. Manual pressing of the stripper leads to egg breaking via the two steel blades of the clamping device and gravity collection of egg liquid (Yin and Yin, 2019). Similarily, another patent discloses an egg-shaped chamber constituted of blades and two suction cups. A single egg is held between the two suction cups and pushed toward the blades with a T-shaped rod leading to eggshell breakage and release of egg liquid (Liang et al., 2018). A cup containing a filter plate and a fixed rod has been patented to drain residual EW and EY from beaten eggs. Briefly, ES is placed on the fixed rod allowing the flow of residual egg liquid toward the perforated filter plate (Gong, 2019). Another ES filter cup can remove contaminating shell fragments from beaten eggs (Bian,2015a,b; Bian,2016a,b). The upper cup contains multiple slits with saw teeth (Bian, 2016a, 2015a) or several rows of sieve holes on the side (Bian, 2016b, 2015b) that allow the smooth flow of beaten liquid egg and prevents the transfer of ES fragments.

#### Separation of Liquid Egg Using Air Pressure

A patented device reports the separation of intact ES from liquid egg by drilling both ends of the egg, followed by blowing out the liquid egg using pressurized air (Wang, 2016a). A related invention describes an ES drilling and aerator device composed of a lifting drill arm, a rotating air drill bit and a spring-pressure aeration device. The lifting drilling device pierces the top and bottom of the egg, and a pressure aeration device is integrated with the air drill bit to introduce air into the inner cavity of the egg to force egg contents through the drilled hole. The ES drilling aerator provides a large amount of intact shell for making ES crafts, with efficient collection of egg contents (Wang M., 2017; Wang, 2016b). A similar patented process involves the creation of air holes at the egg sharp and blunt ends using a clockwork drilling machine. Pressurized air is applied at one air hole leading to force liquid egg through the other hole. The intact ES is suitable for craft work (Wu,2017a,b). Likewise, a hand-held ES drilling and liquid egg separation cylinder is disclosed that works by drilling vent holes at both ends of the egg using a rotating drilling piston. The piston then forces air into the egg through one vent hole to eject liquid egg from the other vent hole, while keeping intact ES suitable for craft work (Yang, 2016; Yang Y., 2017). Another patented process claims the use of an air-assisted egg-shaped egg-breaking apparatus for the separation of EY and EW from ES in which a fixed egg is cracked open by two blades thereby releasing the egg content. The sticky EY is then blown off by compressed air and ejection of the ES by a moving vane (Zhang M., 2018). To produce intact ES suitable for craftwork, an ES rotary electric drilling and inflatable liquid egg extractor has been patented, which rapidly rotates the egg to mix its internal egg contents for their easy removal by forcing air through a hole made by a motor-driven drill (Dong et al., 2018).

A patented process disclosed the use of a device for simultaneous egg placement and ES separation that involves pinhole puncturing and extraction of liquid egg contents via a suction needle tube. ES is automatically separated after liquid egg removal (Yan et al., 2018). Similarily, a patented process involving ES piercing and egg liquid suction has been disclosed involving buffering and suction mechanisms. After penetrating the ES and sucking out egg contents, the buffering component effectively avoids piercing the bottom of the egg (Yao et al., 2017). Another automatic and cost-effective egg shelling device intended for the food industry is composed of an egg sheller box, liquid egg tank, and ES collection tank. High pressure is created inside the sheller box leading to egg collapse and flow of the liquid egg into the liquid egg tank, while the ES accumulates in the ES tank (Wu, 2018).

### Separation of Eggshell From Eggshell Membrane

The ESM is a cross-linked fibrous meshwork that lines the ES and is composed of hundreds of bioactive proteins that are a biomaterial resource distinct from the ES mineral (Ahmed et al., 2019a). Several inventions have been patented to separate ES from ESM, using proteolytic enzymes (Liang et al., 2017), or based on differences in the physical properties of ES and ESM (Zhao et al., 2015; Wang, 2016c; Chen J. et al., 2019; Sun et al., 2019).

**FIGURE 6:**
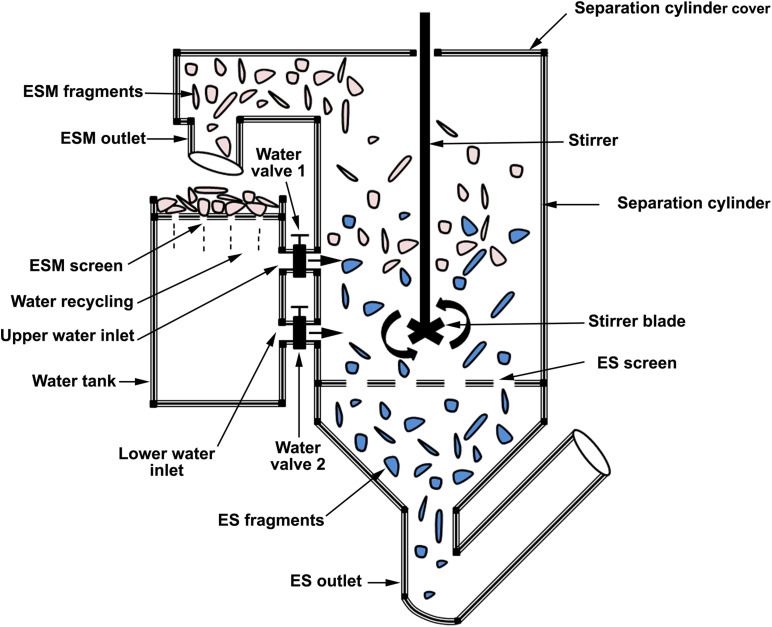
Schematic presentation of patent CN103340441B describing a method to separate ES from ESM.

A patent claims a method to recover ES and ESM from ES waste through enzymatic hydrolysis using kiwi protease, bromelain, ficin, papain, and pepsin or ginger protease. ES and ESM are recovered from the dried solid and liquid phase, respectively. The recovered ES powder can replace existing CaCO_3_ fillers in thermoplastic material (Liang et al., 2017). Another ES separation patent feeds ES fragments through a series of vertical and horizontal wave counter-rotating drums. As the inner membrane of the dried ES has strong toughness and tensile resistance, it resists vertical bending and extrusion, while the fragile ES is easily broken into fine particles. The ESM flows out of the screen outlet while fine ES powder flows out of the chute outlet (Wang, 2016c). A patent discloses ES separation equipment, which comprises a cylinder containing ESM outlet (cylinder top segment), ES outlet (cylinder bottom segment), water inlet (cylinder middle segment), screen (top of ES outlet), and stirring device (cylinder center). ES fragments are cleaned, wetted, and crushed in to the cylinder. Distribution and separation of ES from ESM is achieved by the action of the stirring device along with pressurized water introduced through the water inlet. Heavy ES fragments pass through the screen to accumulate in the ES outlet, while the lighter ESM is directed to the ESM outlet (Zhao et al., 2015; Figure 6). Finally, an integrated device is patented for automated ES cutting and ESM extraction for biomedical applications. The ES cutting component divides the egg into two halves by a combination of rotating ring and wire clamping parts. The ESM are detached from the ES halves by friction rods. This invention preserves the protein constituents of ESM, as it is does not use heat in the separation process (Chen J. et al., 2019; Sun et al., 2019).

### Separation of Eggshell From Hatchlings or Cooked Eggs

Other processes have been patented to separate ES from hatchlings (Li et al.,2019a,b) for the broiler sector, and from cooked eggs (Luo, 2016a; Xu Z. et al., 2016; Yin, 2017; Zhao P., 2017; Lee and Lee, 2018; Furui, 2019; Wang, 2019; Zhang, 2019; Zhong, 2019) for the food industry.

#### Separation of Eggshell in the Broiler Sector

An invention discloses a device for separating newly hatched chicks from small ES fragments. The separation line includes a vibrating screen surface, blower, and a material hopper. Hatchlings, unhatched eggs, and ES fragments fall from the hatcher plate to the vibrating screen. The hatchlings along with small ES fragments slip to the lower hatchlings transport line where the ES is wafted upwards to be collected in the material hopper that is connected to a spiral ES crushing conveyer (Li et al., 2019b). Unhatched eggs are separated from big ES pieces based on the weight difference using a secondary separation device constituted of multiple elastic filter strips. These strips block the transfer of lighter ES pieces while allowing passage of heavier unhatched eggs for manual collection. The lighter ES fragments fall in the material hopper (Li et al., 2019a).

#### Separation of Eggshell From Cooked Eggs

A number of inventions are specifically designed to remove eggshell from hard-boiled eggs (Xu Z. et al., 2016; Yin, 2017; Zhao P., 2017; Lee and Lee, 2018; Zhang, 2019; Furui, 2019), partially cooked eggs (Zhong, 2019) or poached eggs (Luo, 2016a). A simple ES-breaking device for use in the household kitchen has been disclosed. It claims to save time and effort, and produces sanitary peeled eggs. The invention is composed of a motor-driven oval bumper with multiple bumps that enclose the egg. The oval bumper moves up and down to break the ES and facilitate egg peeling (Yin, 2017). Another invention disclosure describes a simple egg top cutter with a cylindrical tube connected to a cone shape cutting blade. A spring is located inside the tube that connects the cutting blade to a metallic ball. The metallic ball drops to hit the tube and transfer the force to the blade edge, which cuts the eggshell (Chang, 2016).

A patented process discloses a large-scale ES peeling device composed of a frame, a feeding area, a shell breaking module, a shell peeling module and temporary storage box. An oblique feeding area promotes the arrangement of rows of cooked eggs on a rack that are driven upwards toward the ES crushing module via a mechanized rail. ES crushing module constituted of multiple ES crushing devices located at the invention rear side. The peeling device is constituted of a gear, a belt, and a peeling rod that enables egg shelling after being crushed. The resultant ES are then collected in the temporary storage box located at the bottom of the peeling module, while the peeled egg is collected at the egg collection port (Zhong, 2019). Another invention for shelling eggs consists of an egg storage trough, egg guide groove, a shelling device and a shell pressing plate that are all sequentially and obliquely arranged. Eggs are automatically rolled down under gravity from the egg storage trough via the egg guide groove to the shelling device. The shell plate press down on the egg to break the shell, and the rolling shaft of the shelling device rolls and presses to remove the shell. The shell plate contains a rubber layer to ensure ES breakage without affecting the integrity of egg contents (Xu Z. et al., 2016). Similarily, a patent reports the utilization of an ES peeling machine constituted of a feeding device and peeling drum supported with a spiral plate. The feeding device allows only the entry of two eggs at a time to the peeling drum. The spiral plate drives the eggs from the egg inlet to the outlet, where ES is peeled under the effect of the rotating drum. Peeled eggs then slide out through the egg outlet slot, while the drum is flushed with water through spray holes to remove ES residues (Zhao P., 2017). Another patent discloses an egg sheller composes of an egg feeding device connected to egg stripping device. Eggs are guided via an egg control device and guiding slide plate allowing organized entry of eggs to the stripping device through the egg entry mouth. The stripping device contains egg pushing and stripping rods that peel away the ES (Zhang, 2019). An automatic ES peeling device is disclosed that involves ES breaking by beating the eggs against split plates with an enhanced orthogonal and parallel collision rate that exhibited improved peeling efficiency for the egg sharp and blunt ends compared to the traditional device (Furui, 2019). Furthermore, a patented invention describes a method for the sequential peeling of ES from cooked eggs with coloring of the cooked EW. The ES peeling apparatus includes: a supply hopper, a conveyer, and a pair of peeling rollers. After peeling the EW is colored using food coloring agents (Lee and Lee, 2018).

A patent is based on the use of negative pressure and hot air to facilitate egg shelling. The invention consists of casing, egg rack, negative-pressure fan, hair dryer, negative-pressure base and lid, and arc clamping device. Eggs are arranged in the egg rack and held at the equatorial region by the curve snap component of the arc clamping device. The sharp and blunt ends of each egg are enclosed by a negative pressure base and lid connected to a negative pressure air fan. The negative pressure leads to egg breakage in the presence of hot air (generated by a hair dryer) blown to the arc snap ring (Luo, 2016a). Another invention describes an ES stripping tool composed of piston, air cavity, tapered suckers and pin holes produced by sharpened rods (pinprickers). The pinpricker pierces the ES and ESM without breaking the EW, and a tapered sucker in contact with the ES prevents air leakage. The piston drives air through the pinhole between ESM and EW leading to separation of ES without compromising the integrity of the cooked egg (Wang, 2019).

## Processing of Eggshell

### Eggshell Washing and Sterilization

Bacterial contamination of ES waste is a potential concern, since egg surfaces may be infected by microorganisms such as *E. coli*, *Salmonella enteritidis* (S. *enteritidis*), *Salmonella typhimurium* (S. *typhimurium*), *Pseudomonas aureus* etc. (De Reu et al., 2006). Shell egg contamination with bacteria and dirt can occur at any of a number of steps in the production chain, from the hen reproductive tract to the market shelf. A patented process claims ES disinfection using oregano essential oil of a grade suitable for veterinary and animal husbandry. Oregano oil exhibited antimicrobial activity against Salmonella when applied to the ES surface. The colony count obtained from the ES surface was reduced to zero after oregano essential oil disinfection, which was significant compared to non-treated eggs (Kobayashi et al., 2018).

An invention describes an ES cleaner composed of a handle and cleaning device with scraping and jagged surfaces to remove packed and loose dirt, respectively, from the egg surface (Ye, 2015; Yang G., 2017). A related invention claims to be convenient ES cleaning device to remove feces. It is composed of a water storage tank, an electric water pump, water sprinkler pan, a tray that is arranged between two sliding rails. Eggs arranged on the tray are spray-washed using the sprinkler pan driven by the water pump. This equipment has the advantage of protecting workers hands and introduces a filter to collect solid material washed off the eggs (Zuo et al., 2019). In addition, two patents disclose a machine for automatic decontamination of poultry eggs. It consists of an egg tray installed on a frame, where the tray top is open, while the bottom has a drain hole. On the frame, a brush plate is installed on a rotating device. The spray washing device includes a water supply pipe and a nozzle facing the upper surface of the eggs. This machine has the advantages of strong self-cleaning ability and high decontamination efficiency (Feng, 2015, 2016). Another cost-effective ES cleaning machine with high cleaning efficiency has been disclosed to be used for food processing. It involves clamping multiple evacuated eggs on a rotary machine and spray washing using sprayed water (He, 2019). A similar invention is composed of the same components; however, it contains an additional hole-creating mechanism to provide a multipurpose ES cutting and cleaning invention (Wang, 2018). A different invention describes a device for creating a hole in the shell of the fertilized egg intended for vaccine production. The invention is constituted of a motor-driven rotating grinding head that is heated to allow sterilizing and confined mill cutting at a defined ES location. The generated ES fragments are drained away via an exhaust pipe (Yu, 2019).

Another patent describes a simultaneous ES grinding and sterilization strategy, where ES is mechanically crushed while undergoing heat sterilization at 80–90°C. The crushed ES is then fractionated using a vibratory sieve (0.5 mm pore size), and the larger ES fragments undergo a second grinding cycle (He, 2017). Similarly, a patent describes an ES sterilizing and grinding machine for downstream processing and reuse of agricultural ES waste. The ES is sterilized at 80–90°C and conveyed in a pulverizing chamber before collection using a 200 mesh screen (Zhang et al., 2015).

ES drying has been considered a challenge as the high viscosity of the remaining albumen causes ES fragments to stick to each other and to the inner surface of the drying apparatus, and thus the drying process takes longer. A patent discloses a continuous drying operation at high temperature for automatic sterilization, separation of ESM and continuous drying of ES. A hopper transports the ES fragments through the drying unit from one side with rotated inside by a spiral protrusion for discharge at the other side (Kim et al., 2018). A related invention describes a modification in the ES dehydrator to facilitate the removal of adhering material. The ES dehydrator resembles a regular washing machine and is driven by a motor. The invention aims primarily at reducing the load applied to the rotating shaft of the motor when ES is dehydrated by providing a clutch on the rotating shaft of the motor, and can prevent the rotating shaft of the motor from being damaged by the rotating load (Jo et al., 2018).

### Artistic Carving of Eggshell

ES is an attractive and creative material for carving to produce artwork and decorative items. Thus, many tools including carving pens, sanders, fixing and attaching material have been patented for ES carving (Shang, 2015; Wang and Cui, 2015; Gao and Xiao, 2016; Jiang, 2017; Chao et al., 2018; Zhu et al., 2018; Dai, 2019; Wang and Zhang, 2019; Wang et al., 2019; Zhang et al., 2019a; Zou,2019a,b,c,d,e).

#### Mechanical Carving

A vibrating ES punching device has been disclosed to evacuate egg without compromising the shell to be used for the carving purposes. The punching device creates a reproducible and precise hole in the ES using a vibrator supported by a spring cushions to prevent cracking (Wang et al., 2019). Similarily, an ES punching and cutting device was patented to produce smooth and even cut suitable for food and handicraft applications. A piston rod triggers the hollow punching die to create an even and smooth cut without any cracks (Dai, 2019). Another patent discloses an egg punching device for carving purposes that relies on the coordination of guide ring and coil spring to perforate the ES along with the movement of the piston and pull rod to withdraw the egg contents; thus the EW and EY are retained for other applications (Wang and Zhang, 2019).

A patented process describes an automatic egg carving machine that detects a feedback through a vibration detector and is constituted of egg clamping part, rotating electric knife, and programmable text or pattern to be engraved. The utility can be used to carve eggshells of different avian species (Chao et al., 2018). Similarily, an automatic ES engraving device has been patented which is composed of ES clamping and cutter swing mechanisms. The invention employs a carving knife connected to a steering gear that carves ES of various specifications with high precision and good consistency (Zhu et al., 2018). A patent claims the use of computer numerical control (CNC) machine to carve patterns designed by computer efficiently on the irregular ES surface. The projected path point method is used to generate the projection from the plane engraving processing path to the ES (Gao and Xiao, 2016). A motor-driven ES engraving pen with three rotating switchable cutting knives has been disclosed for ES carving with improved efficiency and reduced processing time (Zou, 2019d). Alternatively, an ES carving sander has been patented to facilitate the polishing of carved ES handicrafts through the sanding of a rapidly rotating egg with a grinding disk (Zou, 2019b).

Interestingly, an ES carving fixture was patented which is composed of an air cylinder, telescopic rod, rotating screw, adjusting and fixing block that can support various sizes of avian eggs. The egg is fixed so that the carver can concentrate on carving to help reducing the probability of egg breakage (Zou, 2019e). In addition, a support bracket was patented for artistic ES carving that can rotate via a motor-driven shaft and bottom-lit with light to enhance the visual effect (Zou, 2019c).

#### Laser Carving

A laser egg carving device has been disclosed which consists of a laser source and rotating suction cup. The egg is held in position using a vacuum-actuated suction cup and engraved using a laser beam. The held egg is rotated and can be moved up and down to maximize the carving precision and through-put (Wang and Cui, 2015). Similarly, a patent discloses a device for avian ES carving that is equipped with a sandblasting seal box, sand spray gun, conveyer belt, egg holder, iron hollow pattern model, and a programmable logic controller. Sand particles sprayed through the pattern model create concave and convex patterns on the shell surface with <1% failure rate and high efficiency for batch processing (Jiang, 2017). An automatic laser carving machine was patented for ES carving intended for ES handicrafts. The invention takes the advantage of spring buffering to better control the strength of the laser-mediated egg carving and to diminish the risk of ES cracking (Zou, 2019a).

#### Other Carving Strategies

A process for portraying poultry ES has been disclosed involving the surface cleaning of ES and application of a wax layer (0.5-1 mm thick). A pattern or text is carved in the wax layer without penetrating the ES layer. The carved wax along with the underlying ES is soaked in vinegar for 4 to 6 h followed by removal of the wax layer revealing the carved patterns in ES surface. The invention is suitable for industrial production (Shang, 2015). Another patented process provides an ES carving method based on artistic sculpture shaping mud, as the ES is highly fragile during carving. A carving layer constituted of sculpting clay is arranged on the chemically-hardened ES surface. Decorative threads embedded between the gaps of shaping mud are used for ES carving (Zhang et al., 2019a).

### Eggshell for Artwork and Decoration

ES fragments can be applied to produce collage decorative paintings through different texturing strategies (Duan and Duan, 2018). A patented invention describes an ES crushing apparatus for producing lacquerware, to produce lacquer crafts from broken or crushed ES. The device has a pipe-shaped handle and a disk-shape rubbery crushing head. The crushing head surface contains multiple metallic protrusions that promote ES crushing when inserted into the ES halves (Choi, 2016). A related patent claims to produce a resilient hard lacquer ES teacup with great heat insulation capacity. The outer layer of the cup is pasted with ES fragments that are arranged to have even gaps, then painted and polished (Zhang Z., 2018). Another patented process describes the creation of garden pots using ES powder mixed with cement, using a bath towel as a shaping material (Calubaquib, 2018). In addition, a colorful coating method was patented for decoration of intact ES. The invention enables the utilization of ES as an ornament (Bao, 2015). Furthermore, an inkjet printing method was patented for ES labeling that employed edible and water-insoluble pigments (Naoshi and Wataru, 2017).

### Eggshell Cutting

An ES cutting device for vaccine production consists of power cord, stainless steel protective cover, U-shaped ceramic tube sleeve enclosing a resistance wire, and a metal heat conduction ring. The flat end of the metal heat-conducting ring is inserted into the U-shaped ceramic pipe sleeve for sufficient heat conduction, and the cutting ring of the metal heat-conducting ring extends out of the U-shaped ceramic pipe sleeve. Heating of the resistance wire heats the metal heat conduction ring through the U ceramic tube sleeve, for ES circular cutting (Li et al., 2016). Three related patents have been disclosed to carry out fine and accurate ES opening and rounding operations with standard round holes without damaging the inner eggshell membrane, thereby obtaining an optimized egg test product using embryonic tissues. The egg is held in position by a speed-controlled rotating ES opening clamping protection device that is then cut by a micro-precise cutting drill tip during egg rotation (Fan et al.,2020a,b, c). To increase the service lifetime of a hand-held ES drill, a method has been patented which uses pressurized gas to prevent friction between the rotating shaft core and the shaft (Xian et al., 2018). Another patent discloses an electric ES opener for laboratory use, based on a rotating grinding wheel connected to a shaft. The device can accurately cut ES and is convenient to use (Chen et al., 2016). An egg topper cutter to improve the hatching rate of transgenic chicken has been patented and consists of a handle, grinding wheel and cotton groove. One end of the handle is provided with grinding wheel, while the other end has a groove to hold a cotton ball soaked in alcohol. The egg surface is disinfected with the alcohol cotton ball, and ground gently using the grinding wheel until ES opening is complete (Xu T. et al., 2016).

A patent discloses an egg scratching device for wine production and involves the transfer of eggs via a conveyer belt to a light weight grinding wheel. The grinding wheel in combination with a connected spring power device triggers the formation of two lateral and longitudinal cracks in the ES without damaging the ESM (Liu et al., 2015). Another invention consists of a cutting device with a gripping body, a cylindrical cutting blade body, and an ultrasonic generator to ultrasonically vibrate the cutting blade body. The rotating and vibrating cylindrical cutting blade cuts a circular opening in the eggshell (Hirai, 2019).

### Eggshell Crushing

**FIGURE 7:**
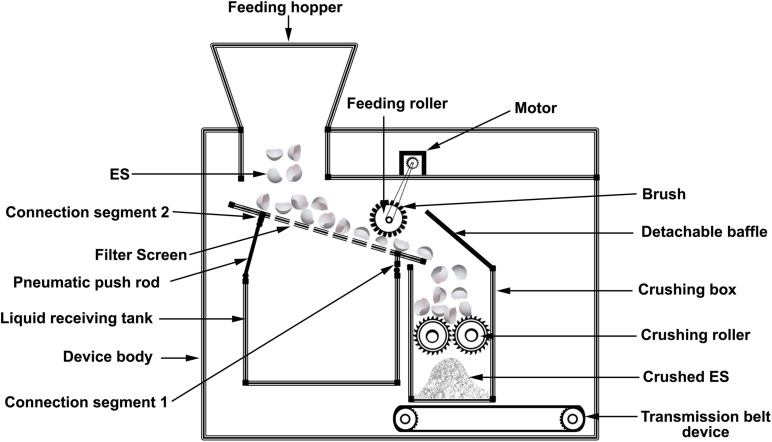
Schematic presentation of patent CN206404840U describing an ES crushing device.

An invention discloses an ES recovery device for confined spaces that enables ES waste recycling. It consists of a workbench, conveyer bed, recycling bins, hydraulic device, directional heat brattice, and absorbing paper. The hydraulic device includes a stretchy convex needle array head. ES falls on the absorbent paper on the horizontal bed conveyor. The needle array head adjusts to the orientation of ES halves, as ES is transferred forward on the conveyer bed, disinfected and collected in the recycling bin. Contaminated air flows from an air outlet (Zhao and Dai, 2019). Another patented model for an ES crushing device consists of a feeding hopper above a liquid collection device. Liquid is filtered through a screen to exclude ES fragments. A feeding roller with a brush pushes the ES to the crushing box, which is equipped with a pair of opposing rollers to crush ES efficiently (Wang D., 2017; Figure 7). Another patented ES crusher involves the loading of ES via a feeding port into a motor-driven crushing box, which contains two pressing blocks driven by a group of gears, telescopic rods, and springs. Crushed ES is then discharged into a collection box (Yang et al., 2019).

### Eggshell Powder

An invention discloses a preparation method for ES powder involving ES cleaning, peeling, drying, pasteurization, and crushing to attain a particle size of ≤ 38 ± 5 μm (Stepanova and Kondratyuk, 2016). Another invention discloses an ES processing device that is constituted of a feed port, crushing box, vibrating sieve, and storage box. The crushing mechanism depends on a spindle and crushing rollers. ES material is fed into the crushing cavity via the feeding port and crushed by the rollers. Crushed ES is then sieved via the vibrating sieve located at the lower end of the crushing box, and fine ES powder collected in the storage box (Qiu, 2018). Similarly, a disclosed patent describes an ES processing device that uses a rotary furnace to prepare ES powder. It includes a rotary furnace, a feeding device, an ES powder pulverizing device and a waste gas treatment device. The feeding device is set at the inlet of the rotary kiln and the ES powder pulverizing device is installed at the outlet of the rotary furnace. The generated waste gas is effectively treated to reduce environmental pollution (Wang et al., 2017). Another patent discloses simultaneous ES and ESM recycling without the need to separate ESM from ES and comprises washing, sterilizing, grinding, hydrolysis, and separation steps. The sterilized ES is crushed to increase the specific surface area and undergoes enzymatic hydrolysis using proteases (e.g., kiwi protease, bromelain, ficin, papain, pepsin, or ginger protease). The hydrolyzed ES material then undergoes solid-liquid phase separation using suction filtration, pressure filtration or centrifugal separation. The solid phase substances are freeze-dried to obtain ES powder, while the liquid phase substances are freeze-dried to obtain ESM powder (He and Li, 2017).

### Intact Eggshell

A patented method describes a method for food cooking using emptied eggshell. Egg contents are removed through a hole made in the ES, and food ingredients are packed into the emptied egg. The ES hole is sealed with environmentally friendly food grade adhesive flour dough to prevent leakage of the filled food, followed by cooking (steaming, boiling, or baking) (Kang, 2017). Similarily, emptied ES was patented as an appealing and creative container to cook mince fillings (Sun and Sun, 2015), to prepare Creme Brulee (Liu H. et al., 2019), to package wine and beverage (Wu, 2016), and bread (Han, 2018). Sterilized ES was also patented as a green and economic solution to cultivate vegetables (Zhao S., 2017).

## Conclusion and Prospects

The solid ES waste generated by breaking plants is often discarded in landfill sites or utilized for various low-value applications, including lime substitute, animal feed, and fertilizer. In addition to the environmental impact, ES waste disposal in landfill sites according to local environmental regulations is associated with the loss of valuable calcium carbonate and bioactive protein constituents. However, ES combines several advantages making it an ideal raw material for developing biomedical, chemical, engineering, and environmental technologies (Ahmed et al., unpublished). This review summarizes different recently patented engineering technologies for the screening, separation, and processing of ES. These technologies are considered to be sequential approaches to enable the valorization of ES waste. Screening technologies include eggshell surface spots and glossiness, eggshell cracks, and mechanical properties, along with detecting chicken breed and surface bacterial count. Collection and separation technologies describe separation strategies of ES from egg white (EW), egg yolk (EY), liquid egg, eggshell membrane (ESM), hatchlings, and cooked egg. Processing of ES involves washing and sterilization along with carving, cutting, crushing, and pulverization technologies. The washing and sterilization technologies are the primary processing approaches to generate high-quality ES for value-added applications. For industrial-scale utilization of ES, rapid and accurate screening of ES surface qualities based on computerized analysis of light and sound waves are significant approaches. Exploitation of rotational forces for efficient breaking and separation of ES from egg liquid compartments is a rational strategy. Processing of high-density ES and low-density ESM in the presence of pressurized water enables efficient and simultaneous separation, washing, and cleaning of ES. Simultaneous ES pulverizing and drying via rotary furnace allows optimum packaging and storage. For future research directions, the exploitation of pulverization strategies to obtain tailor-sized ES material and optimization of ES as an immobilization carrier are novel ventures. The dramatic increase in the number of innovative screening, separation, collection, and processing technologies reflects investor and researcher interest in creating profit from low-value ES byproducts and is an essential step to diversify its applications.

## Author Contributions

All authors approved the manuscript content and warrant that this review manuscript is not under consideration for publication elsewhere. All coauthors have contributed in a significant way to the final format of the review.

## Conflict of Interest

The authors declare that the research was conducted in the absence of any commercial or financial relationships that could be construed as a potential conflict of interest.
